# Editorial: Regulation of Immunity to Parasitic Infections Endemic to Africa

**DOI:** 10.3389/fimmu.2020.01159

**Published:** 2020-06-12

**Authors:** Faith Osier, Jude E. Uzonna

**Affiliations:** ^1^Centre for Infectious Diseases, Heidelberg University Hospital, Heidelberg, Germany; ^2^KEMRI Wellcome Trust Research Programme, Centre for Geographical Medicine Research, Kilifi, Kenya; ^3^Department of Immunology, Rady Faculty of Health Sciences, Max Rady College of Medicine, University of Manitoba, Winnipeg, MB, Canada

**Keywords:** parasitic infections, immune response, malaria, trypanosomiasis, leishmaniasis, toxoplasmosis, fascioliasis

Parasitic diseases that affect both humans and animals are major causes of morbidity and mortality across the world, and particularly in Africa where they are endemic (1). They are often closely related to poverty and with the exception of malaria, are considered neglected because they receive relatively lower treatment and research funding in comparison to HIV/AIDS and tuberculosis (2). However, their combined socioeconomic impact in sub-Saharan Africa is comparable to that of tuberculosis and HIV/AIDS (3). Parasitic infections can be transmitted across geographical barriers and warrant global attention (4). Environmental factors such as humidity and warm temperature promote year-round development of parasites and insect vectors, thereby sustaining transmission. In addition, poor sanitary living conditions and overcrowding promote disease transmission.

A better understanding of parasite biology, pathology, immunology, and parasite-host interactions has resulted in better therapeutics and management strategies that have significantly improved patient outcomes. Unfortunately, many parasites develop resistance (5) thereby thwarting the effectiveness of therapeutic strategies. Parasite genomes encode diverse proteins that interact with the host immune system in a dynamic and complex fashion that leads to evasion of protective anti-parasitic mechanisms (6). The articles published in this Research Topic provide insights on current advances in research on parasite biology, host immune responses to parasites and novel therapeutic strategies for parasitic infections such as malaria, trypanosomiasis, leishmaniasis, toxoplasmosis, and fascioliasis.

Several original articles in this Research Topic focus on antigen-specific immune responses to the malaria parasite. Aniweh et al. demonstrated that antibodies against the newly characterized *Plasmodium falciparum* merozoite associated protein (PfMAAP) potently prevented the infection of red blood cells by *P. falciparum* merozoites and were associated with a reduced risk of malaria in humans. Kivisi et al. showed that maternal-derived antibodies against variant surface antigens of *P. falciparum* imposed a selection pressure on the parasites and was associated with reduced parasitemia in infants. This latter study supports a potentially new mechanism for maternal-induced immunity against malaria in the early stages of infancy. The transmission of *P. falciparum* from mosquitoes to humans can be significantly reduced by targeting the transmission stage (gametocyte) of the parasite and this has energized the search for transmission blocking vaccine (TBV) candidates. Although significant strides have been made against *P. falciparum* infection, TBV development against *P. vivax* has not been adequately explored.

*P. vivax* is the leading cause of malaria in Latin America. To investigate the potential of TBVs against *P. vivax*, Tentokam et al. analyzed the seroprevalence of antibodies against Pvs230D1M in naturally infected individuals in Brazil and Cambodia. This antigen is located in gametes of *P. vivax* and few polymorphisms have been reported worldwide. They detected similar levels of antibody responses to Pvs230 in these distinct populations from regions with differing transmission intensities, highlighting its potential as a TBV. A separate study focused on the significant role of T-cells in immunity against malaria. Frimpong et al. compared the expression of markers of T-cell inhibition and senescence in healthy children to those with symptomatic or asymptomatic malaria, and have made striking differences observations. Children with symptomatic malaria expressed higher levels of inhibitory and senescent markers on their T cells compared to asymptomatic patients and healthy controls. This suggests that effector T cell function may be impaired in patients with symptomatic malaria and could result in elevated parasitemia.

Parasites belonging to the genus *Trypanosoma* are transmitted by the tsetse fly and cause “sleeping sickness” in humans and wasting disease in livestock. Although the disease currently affects thousands of people and millions more are at risk, trypanosomiasis in animals is more prevalent and poses serious agricultural and economic problems in affected regions. The production of proinflammatory cytokines by macrophages is essential for resistance. However, trypanosome-induced intracellular signaling pathways that lead to macrophage activation and production of proinflammatory cytokines remain poorly defined.

Kuriakose et al. showed that the production of proinflammatory cytokines (IL-6, IL-12, and tumor necrosis factor alpha, TNF-α) by macrophages during *Trypanosoma congolense* infection involves the activation of mitogen-activated protein kinase (MAPK) and signal transducer and activator of transcription (STAT) signaling pathways. They further showed that toll-like receptor 2 (TLR2) and the adaptor molecule, myeloid differentiation primary response 88 (MyD88), are critically involved in this process and that deficiency of MyD88 and TLR2 leads to impaired cytokine production and acute death of *T. congolense*-infected compared to resistant mice. This study expands our knowledge about the signaling pathways involved in the immune response to *T. congolense*, a major etiologic agent of African trypanosomiasis in livestock. Campbell et al. proposed a potential mechanism of immune evasion by *T. brucei*, another species of African trypanosomes. They demonstrated that aromatic ketoacids secreted by *T. brucei brucei* promote the expression of heme oxygenase (a stress protein) and inhibit the production of proinflammatory cytokines in macrophages and glia cells. Morenikeji et al. utilized an *in-silico* approach to identify conserved miRNAs that regulate the expression of genes involved in the immune response during bovine *Trypanosoma* infection. They proposed the use of miRNAs as biomarkers for diagnosis, drug design, targeting and treatment of bovine trypanosomiasis.

Leishmaniasis is endemic in North, East and Central African countries. Globally, nearly 30,000 deaths occur annually and over 1 billion people are at risk of infection. The production of proinflammatory cytokines and T-helper cell responses both play a major role in immunity to *Leishmania* infection. Münck et al. showed that during the early stages of *Leishmania major* infection, the transcription factor, aryl hydrocarbon receptor (AhR), was significantly upregulated in murine lesion-associated macrophages and was associated with increased production of proinflammatory cytokines such as tumor necrosis factor (TNF). The local administration of an AhR agonist to susceptible BALB/c mice resulted in reduced disease severity as well as a decreased Th2 response and parasite burden, suggesting a critical role of this pathway in resistance. McFarlane et al. showed that in contrast to findings in BALB/c mice possessing a global knock out of IL-4Rα, deficiency of IL-4Rα on either CD4^+^ T cell or total T cells in BALB/c mice did not significantly affect their resistance to *L. donovani* infection. This study suggests that the observed protective role of IL-4 and IL-13 in *L. donovani*^1^ infected IL-4Rα global knockout mice (7, 8) is not mediated by IL-4Rα-responsive T cells.

Other parasitic infections highlighted in this Research Topic include fascioliasis and toxoplasmosis. Chen et al. examined the role of Cathepsin B, a lysosomal protease of *Fasciola* and assessed its effects on peripheral blood mononuclear cells (PBMCs) derived from goats. They demonstrated that recombinant^2^ *Fasciola gigantica* cathepsin B (rFgCatB) protein decreases the viability of PBMCs *in vitro* but increases their expression of nitric oxide and cytokines such as IL-4, IL-2, IL-10, IFN-γ, TGF-β, and IL-17. He et al. investigated the changes in gene expression of porcine tissues following *Toxoplasma gondii* infection. In addition to tissue-specific transcriptional changes, they observed an elevation in the expression of genes related to the immune response, while the expression of genes involved in metabolic pathways such as lipid metabolism was reduced.

This Research Topic also includes review articles on immune responses to malaria and trypanosomiasis and the role of macrophage migration inhibitory factor (MIF) on the immune response to parasitic infections. Muthui et al. conducted a systematic review of studies in African populations which examined the antibody^3^ response to Pfs230 and Pfs48/45 antigens, expressed by *Plasmodium* gametocytes. Although antibodies to both antigens were detected in most studies reviewed, there was significant heterogeneity between study results due to different methods used. This underscores the importance of standardized protocols for conducting scientific studies. Kimenyi et al. critically reviewed the dynamics of host-parasite immune interactions in patients with asymptomatic malaria and proposed the use of RNA sequencing to investigate the immune response during asymptomatic malaria infection. In their review, Onyilagha and Uzonna thoroughly examined the factors that affect the immune response to African trypanosomiasis and discussed several immune evasion strategies adopted by this parasite. In particular, they focused on factors that regulate immunity and immunosuppression during *T. congolense* infection and highlighted the possibility that these immunosuppressive factors could aid the evasion of host immune defenses by the parasites.

Ghosh et al. reviewed the effect of macrophage migration inhibitory factor (MIF) secreted by parasites on the host immune response to parasitic infections. The authors also highlighted the potential of MIF as a therapeutic target against parasitic infections.

Collectively, the articles in this Research Topic highlight the complexities of parasite-host interactions, immune responses and disease pathology in parasitic infections. In addition to a better mechanistic understanding of the biology and pathogenesis of parasitic diseases, insights obtained from the articles published in this special issue may contribute to the development of more targeted and effective strategies to control parasitic infections. Importantly, therapeutic agents such as antibodies to *Plasmodium* antigens which promote beneficial host immune responses against parasites may serve as potential treatment options to control disease symptoms and severity. Thus, this is a much-needed and important area of research to increase the armory of tools against parasitic diseases endemic to Africa.

## Author Contributions

All authors listed have made a substantial, direct and intellectual contribution to the work, and approved it for publication.

## Conflict of Interest

The authors declare that the research was conducted in the absence of any commercial or financial relationships that could be construed as a potential conflict of interest.

## Footnotes

^1^Vector-borne Diseases. Available online at: https://www.who.int/news-room/fact-sheets/detail/vector-borne-diseases (accessed March 12, 2020).

^2^WHO. *Environmental Factors Influencing the Spread of Communicable Diseases*. *WHO* Available online at: https://www.who.int/environmental_health_emergencies/disease_outbreaks/communicable_diseases/en/ (accessed March 21, 2020).

^3^gohrd_analysis_leishmaniasis.pdf. Available online at: https://www.who.int/research-observatory/analyses/gohrd_analysis_leishmaniasis.pdf (accessed April 4, 2020).
